# Sex-specific issues of central and peripheral arginine-vasopressin concentrations in neurocritical care patients

**DOI:** 10.1186/s12868-022-00757-1

**Published:** 2022-11-24

**Authors:** A. H. Podtschaske, J. Martin, B. Ulm, B. Jungwirth, S. M. Kagerbauer

**Affiliations:** 1 Department of Anaesthesiology and Intensive Care Medicine, School of Medicine, Technical University of Munich, Munich, Germany; 2 Department of Anaesthesiology and Intensive Care Medicine, School of Medicine, University of Ulm, Albert-Einstein-Allee 23, 89081 Ulm, Germany

**Keywords:** Arginine-vasopressin, Neurocritical care, Brain injury, Sex differences

## Abstract

**Background:**

Arginine-Vasopressin (AVP) is a nonapeptide that exerts multiple functions within the central nervous system and in the blood circulation that might contribute to outcome in critically ill patients. Sex differences have been found for mental and physical effects of AVP. For example, stress response and response due to hemorrhage differ between males and females, at least in animal studies. Data on humans -especially on AVP within the central nervous system (CNS)—are scarce, as cerebrospinal fluid (CSF) which is said to represent central AVP activity, has to be collected by means of invasive procedures. Here we present data on 30 neurocritical care patients where we simultaneously collected blood, CSF and saliva to analyze concentrations in the central and peripheral compartments.

**Patients and Methods:**

30 neurocritical care patients were included (13 male, 13 postmenopausal female, 4 premenopausal female) with a median age of 60 years. CSF, plasma and saliva were obtained simultaneously once in each patient and analyzed for AVP concentrations. Correlations between the central compartment represented by CSF, and the peripheral compartment represented by plasma and saliva, were identified. Relations between AVP concentrations and serum sodium and hematocrit were also determined.

**Results:**

In the whole patient collective, only very weak to weak correlations could be detected between AVP plasma/CSF, plasma/saliva and CSF/saliva as well as between AVP concentrations in each of the compartments and serum sodium/hematocrit. Regarding the subgroup of postmenopausal females, a significant moderate correlation could be detected for AVP in plasma and CSF and AVP CSF and serum sodium.

**Conclusion:**

Absolute concentrations of AVP in central and peripheral compartments did not show sex differences. However, correlations between AVP plasma and CSF and AVP CSF and serum sodium in postmenopausal females indicate differences in AVP secretion and AVP response to triggers that deserve further examination.

## Introduction

Arginine vasopressin (AVP) is a nonapeptide which is synthesized in the hypothalamus and travels via axonal pathways to the posterior pituitary lobe. When it is released into the bloodstream, it exerts so-called peripheral effects such as vasoconstriction, reabsorption of water from the kidney and blood-pressure regulation [1]. Moreover, it can be released via a somato-dendritic pathway within the brain where it modulates behavioral patterns like aggression or pair bonding and contributes to stress-related behavior and anxiety [2].

AVP can be detected in various body compartments, including blood, cerebrospinal fluid, and saliva. The blood–brain barrier is assumed to be impermeable for endogenous AVP, and AVP secretion within the central nervous system (CNS) and in the bloodstream are not necessarily coupled. Therefore, it cannot generally be assumed that changes of AVP concentrations in cerebrospinal fluid (CSF) and other body fluids like blood, saliva or urine always move in the same direction [3, 4]. AVP is regarded a potential biomarker for posttraumatic stress disorder or anxiety [5, 6], which may contribute to physical well-being and long-term-outcome in critical care patients. Furthermore, it is involved in osmoregulation and fluid balance [7], being crucial for acute treatment in critical care.

Sex differences in the AVP system have been found in animals as well as humans concerning central and peripheral properties of AVP which also play a role in critical care. For example, a positive correlation of plasma AVP concentration and distress has been found in men, but not in women [8]. In animal studies, hypothalamic neuropeptide expression as part of a response to chronic stress differs between male and female mice [9]. As far as peripheral AVP actions are concerned, female rats showed a more pronounced AVP response due to hemorrhage whereas plasma osmolality was lower than in males [10].

Taken together, there is strong evidence for sex differences in the secretion and effects of AVP within the CNS and periphery in animal models. However, data on humans are scarce and since CSF can only be obtained invasively, blood or even saliva concentrations are often used as surrogate parameters.

The aim of our study was to analyze AVP concentrations in the two peripheral compartments blood and saliva as well as in the central compartment represented by CSF to detect correlations of neuropeptide concentrations that allow to infer from peripheral to central concentrations in neurocritical care patients with ventricular drain. In addition to the whole patient collective, we analyzed men and pre- and postmenopausal women separately to detect differences between groups. Furthermore, we correlated AVP concentrations in the three compartments blood, CSF and saliva with the routinely collected laboratory parameters serum sodium and hematocrit and analyzed for deviations between sexes.

## Patients and methods

The study was approved by the institutional ethics committee of the Medical Faculty of the Technical University of Munich (Project Number: 282/14). We prospectively analyzed simultaneously taken blood, CSF and saliva samples from 30 consecutive neurocritical care patients between October 2018 and February 2019. 13 patients were male, 13 female postmenopausal and 4 female premenopausal. Median age was 60 years (38–86 years). The inclusion criteria were: adult, hemodynamically stable patients (> 18 years) with external ventricular drainage and existing arterial or central venous access for noninvasive collection of blood and CSF. Either the patient himself or his legal representative had to give written informed consent to participate in the study.

Patients with acute infections, including acute CNS infections, patients with acute hemorrhages requiring transfusion, and patients showing signs of diabetes insipidus or hypovolemia were excluded.

14 patients suffered from aneurysmal SAH, 8 from brain trauma, 3 from brain tumor, 4 had a non-traumatic cause of intracranial hemorrhage and 1 patient suffered from hydrocephalus as a late consequence of a CNS infection. Two patients died during the hospital stay. For each patient, we recorded blood hematocrit and serum sodium from the respective day’s routine lab examination at the timepoint nearest to AVP sample collection and the patients’ Glasgow Outcome Score at discharge from the ICU. Patient data are shown in Table 1.

**Table 1:** Patient core data

Age [years]	Sex	Sodium [mmol/l]	Hematocrit [%]	GOS	AVP Plasma [pg/ml]	AVP CSF [pg/ml]	AVP Saliva [pg/ml]
40	F	139	31.8	4	3.59	2.93	1.88
49	F	132	31.6	3	2.85	4.11	1.52
50	F	141	27.1	3	7.13	3.29	0.92
51	F	144	22.7	3	2.10	2.18	1.71
55	F	135	26.3	3	2.25	3.55	2.61
56	F	139	24.8	3	2.10	2.79	1.86
57	F	144	27.6	3	1.22	3.00	3.99
58	F	140	34.4	2	1.36	1.87	4.66
61	F	138	35.1	5	1.74	2.46	1.76
66	F	128	24.2	3	3.27	3.95	1.19
69	F	145	38.2	3	3.01	3.02	1.01
75	F	138	24.1	2	2.49	3.11	2.09
76	F	145	28.3	1	2.93	1.95	1.68
76	F	138	29.2	3	7.18	3.91	2.96
79	F	137	23.0	3	3.09	5.01	1.51
80	F	136	27.9	3	2.22	3.73	2.36
86	F	142	26.9	1	2.29	2.75	1.27
38	M	144	27.6	2	5.02	4.91	1.35
42	M	144	22.3	3	3.22	1.99	0.90
42	M	139	28.8	2	4.11	2.99	0.91
50	M	141	33.6	2	3.10	3.18	1.41
53	M	142	33.0	3	1.09	6.51	1.04
59	M	139	37.4	5	3.12	3.14	2.42
59	M	135	29.9	3	2.33	2.10	1.72
66	M	136	30.2	2	2.14	4.40	1.67
78	M	142	23.6	3	1.14	2.68	1.03
80	M	133	31.2	2	2.61	3.87	1.09
80	M	141	28.4	3	2.42	3.71	1.25
81	M	139	25.1	2	5.40	1.62	2.90
85	M	154	24.3	2	3.45	8.54	4.52

*GOS* Glasgow Outcome Score at discharge from ICU

CSF, blood and saliva samples were obtained simultaneously once in each patient. All samples were drawn during regular day duty at the intensive care unit between 07:30 and 16:30.

We took blood from a pre-existing arterial line and CSF from the ventricular drain in pre-chilled EDTA-Tubes. Saliva was obtained by means of a cotton swab (Sarstedt^©^Salivette) that was placed in the patient’s cheek pocket for 1 min. Samples were put on ice and transported to the laboratory immediately. After centrifugation for 10 min at 1300 *g* and 4 °C, aliquots were stored at − 80 °C until analysis. Plasma, CSF and saliva were treated identically and analyzed as described before [11]. A highly sensitive and specific radioimmunoassay was performed by an external laboratory (RIAgnosis, Sinzing, Germany). Samples were extracted using LiChroprep Si60 (Merck, Darmstadt, Germany) heat-activated for 3 h. 20 mg of LiChroprep Si60 in 1 mL of distilled water was added to the samples, mixed for 30 min, washed twice with distilled water and 0.01 mol/l HCl, and eluded with 60% acetone. Extraction resulted in a recovery of 85–90% for plasma, CSF and saliva. 50 µl of assay buffer solution and 50 µl of anti-AVP antibodies raised in rabbits were applied to the extracted samples followed by a 1-h preincubation period. After that, 10 µl of ^125^I-labelled AVP (Perkin Elmer, Boston, MA, USA) was added. The method is described in detail by Landgraf et al. [12].

Assay sensitivity was about 0.5 pg, cross-reactivities with related peptides were < 0.7%, and intra- and interassay-variabilities were < 10% [13].

Statistical analysis was performed using R, version 4.2.1 (the R foundation for statistical computing, Vienna, Austria). Data were analyzed for normal distribution by Shapiro–Wilk test. Differences in hormone concentrations in the three compartments as well as differences in AVP concentrations, serum sodium and hematocrit between sexes were determined by Friedman test and post-hoc Wilcoxon signed-rank test and Bonferroni correction where appropriate. Correlations of AVP concentrations in the different compartment and correlations of AVP and serum sodium concentrations as well as AVP concentrations and hematocrit were assessed with Spearman’s rank correlation coefficient. In this article, we describe correlation coefficients < 0.1 as “very weak” which means negligible, coefficients between 0.1 and 0.4 as “weak” and coefficients between 0.4 and 0.7 as “moderate” [14]. Only correlation coefficients of 0.7 or higher indicate a strong relationship by means of a firm rule [15]. Kruskal–Wallis-Test was performed to assess relations between AVP levels and patient outcome. P < 0.05 was considered statistically significant.

## Results

**Fig. 1:**
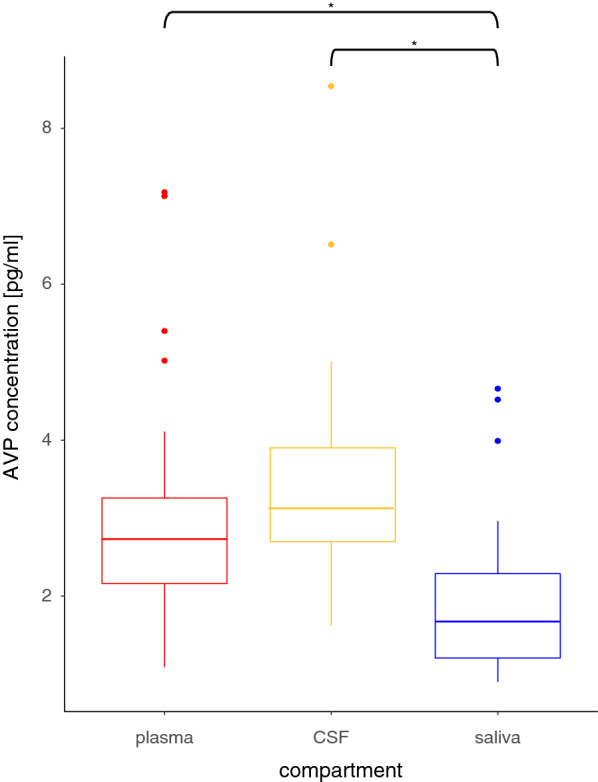
Concentrations of AVP in plasma, CSF and saliva in the whole patient collective. *p < 0.01

All patients showed detectable AVP concentrations in each of the three compartments blood, CSF and saliva which are compatible with those described in the literature [13, 16, 17]. As concentrations of AVP in the three compartments were not normally distributed, Friedman test was performed which showed significant differences between groups. Post-hoc-tests showed that AVP saliva concentrations (median 1.67 pg/ml, IQR (interquartile range) 1.21–2.29 pg/ml) were significantly lower than plasma (median 2.73 pg/ml, IQR 2.16–3.26 pg/ml) and CSF (3.13 pg/ml, IQR 2.7–3.9 pg/ml) concentrations (p < 0.01) whereas there was no significant difference between plasma and CSF concentrations (Fig. 1). Absolute AVP concentrations of male and female patients did not differ significantly in any of the compartments. The parameters age, GOS, serum sodium and hematocrit also showed no difference between sexes.

**Fig. 2:**
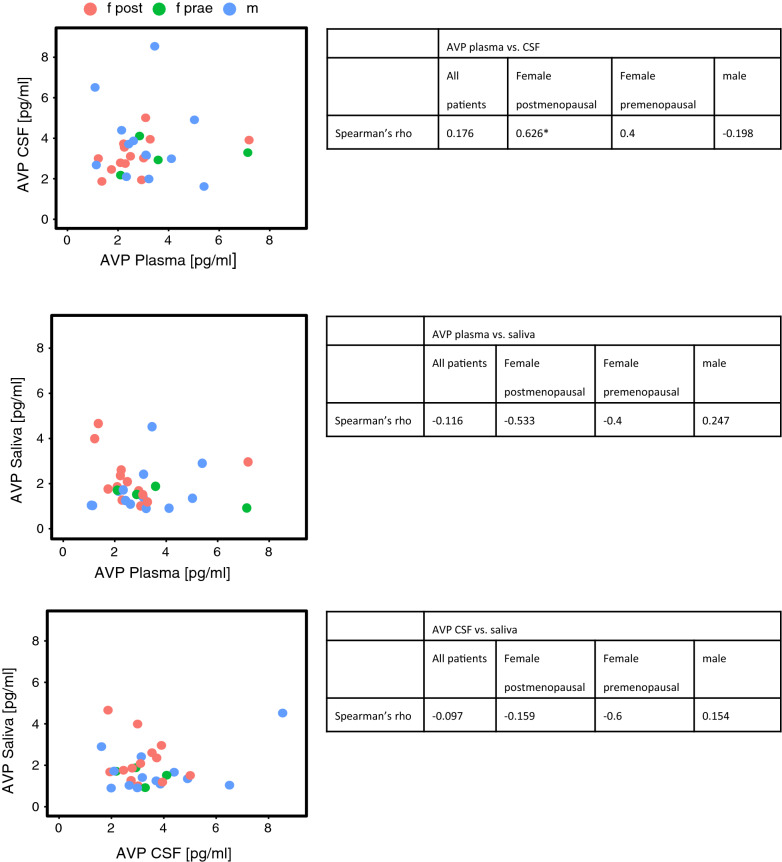
Relations of AVP in CSF, plasma and saliva in the whole patient collective and the subgroups female postmenopausal (f post, red), female premenopausal (f pre, green) and male (m, blue). 95%-confidence intervals are given in square brackets. *p < 0.05

Regarding the whole patient collective, correlation coefficients of AVP plasma/CSF, plasma/saliva and CSF/saliva were weak to very weak. AVP in none of the three compartments correlated significantly with serum sodium or hematocrit either. When analyzing the subgroups, it was noticeable that a significant moderate correlation of AVP in plasma and CSF could be detected in postmenopausal women. A moderate, but not significant correlation was found in this group for AVP in plasma and saliva. Scatterplots of AVP concentrations in plasma and CSF, plasma and saliva and saliva and CSF are shown in Fig. 2.

Concentrations of serum sodium and hematocrit showed very weak correlations with AVP concentrations in the three compartments plasma, CSF and saliva. Again, there was an exception in the group of postmenopausal females where AVP CSF showed moderate negative correlations with serum sodium and hematocrit. Correlation coefficients and p-values are depicted in Table 2.

**Table 2:** Correlations of AVP concentrations and serum sodium and hematocrit

	All patients		Female postmenopausal		Female premenopausal		male	
	Spearman’s rho	p-value	Spearman’s rho	p-value	Spearman’s rho	p-value	Spearman’s rho	p-value
AVP CSF vs. Na	− 0.237 [− 0.554 to 0.140]	0.208	− 0.694* [− 0.913 to − 0.164]	0.008	− 0.8 [− 0.998 to 0.819]	0.333	0.283 [− 0.329 to 0.727]	0.349
AVP plasma vs. Na	0.002 [− 0.359 to 0.362]	0.993	− 0.23 [− 0.697 to 0.375]	0.45	− 0.2 [− 0.975 to 0.944]	0.917	0.2 [− 0.400 to 0.680]	0.513
AVP saliva vs. Na	− 0.135 [− 0.474 to 0.238]	0.476	− 0.066 [− 0.596 to 0.504]	0.829	0 [− 0.961 to 0.961]	1	− 0.153 [− 0.651 to 0.438]	0.619
AVP CSF vs. Hkt	0.051 [− 0.316 to 0.404]	0.791	− 0.522 [− 0.845 to 0.081]	0.071	0.4 [− 0.924 to 0.986]	0.75	0.286 [− 0.326 to 0.729]	0.344
AVP plasma vs. Hkt	− 0.063 [− 0.415 to 0.304]	0.739	− 0.22 [− 0.691 to 0.383]	0.47	0.4 [− 0.924 to 0.986]	0.75	− 0.313 [− 0.744 to 0.301]	0.297
AVP saliva vs. Hkt	0.063 [− 0.304 to 0.414]	0.74	0.132 [− 0.454 to 0.638]	0.669	0.4 [− 0.924 to 0.986]	0.75	0.198 [− 0.401 to 0.678]	0.517

*Na* sodium, *Hkt* hematocrit

Due to the small size of the subgroups, statistical significance was only evident from a correlation coefficient ρ > 0.69 (*p < 0.01)

Confidence intervals (given in square brackets) are very wide throughout, however, in postmenopausal females, a strict negative correlation can be assumed in AVP CSF vs. serum sodium concentrations

AVP concentrations in CSF, plasma and saliva did not differ significantly between the outcome groups defined according to GOS (Table 3). Due to the small group size, we did not analyze for sex differences, however, GOS at discharge from ICU did not differ significantly between males and females.

**Table 3:** AVP concentrations in plasma, CSF and saliva by GOS outcome groups

GOS	No. of patients	AVP Plasma	AVP CSF	AVP Saliva
		(median [IQR])	(median [IQR])	(median [IQR])
1	2	2.61 [2.45, 2.77]	2.35 [2.15, 2.55]	1.48 [1.37, 1.58]
2	9	3.10 [2.49, 4.11]	3.18 [2.99, 4.39]	1.67 [1.35, 2.90]
3	16	2.38 [2.10, 3.12]	3.42 [2.76, 3.92]	1.52 [1.04, 1.98]
4 + 5	3	3.12 [2.43, 3.35]	2.93 [2.70, 3.04]	1.88 [1.82, 2.15]
p		0.618	0.319	0.500

*GOS* Glasgow Outcome Score

## Discussion

In our patient collective of neurocritical care patients, we found only weak correlations of AVP concentrations in the central and peripheral compartments. A significant correlation between central or peripheral AVP and hematocrit and sodium concentrations in blood could not be demonstrated either. The picture is different in postmenopausal women: Here, a significant positive correlation between AVP in plasma and CSF could be detected. Furthermore, we found moderate inverse correlations between AVP in CSF and sodium and AVP in CSF and hematocrit in this subgroup with the correlation between AVP CSF and sodium being statistically significant. Outcome was not significantly related to either central or peripheral AVP concentrations.

The nonapeptide AVP exerts multiple functions. AVP secretion within the central nervous system is considered crucial for maintaining a mental balance, and disturbances in the central neuropeptide system, in which AVP plays an important role, may contribute to the development of psychiatric issues like post-traumatic stress disorder [5]. Especially in brain trauma, intracerebral AVP is said to exert proinflammatory effects [18].

In the periphery, AVP mediates the maintenance of blood pressure and water and electrolyte homeostasis; additionally, it is involved in the endocrine stress response [7]. Stimuli for AVP secretion are fever, pain and psychological stress. Therefore, blood AVP concentrations are considered a potential biomarker for poor psychological and physical outcome of intensive care patients [19]. Due to these facts, AVP is of increasing interest in critical care medicine. In the context of the Covid-pandemic it has been shown that arginine-vasopressin is increased in patients with fever and dehydration and associated with hyponatremia and inflammatory disorders. Therefore, it might contribute to the development of complications [19]. Already some years before, it was found that hyponatremia and resulting elevated AVP blood concentrations are correlated with poor outcome and mortality in patients with community acquired pneumonia [20].

AVP is detectable in various body fluids such as blood, saliva, urine and cerebrospinal fluid. However, the majority of studies do not distinguish between central and peripheral AVP concentrations. As data on CSF are rare, blood concentrations are mostly used as a surrogate and the exact relationship between the concentrations of the nonapeptide within the CNS and in the periphery is still unclear. Although separate central and peripheral secretion mechanisms are assumed, blood or saliva are used for many questions due to their ease of extraction.

In a previous work on a comparable—not identical—patient population, our group could already show for oxytocin, which is closely related to AVP, that blood concentrations correlate only weakly with CSF concentrations [11]. In the case of oxytocin, however, there was a moderate to strong correlation between saliva and CSF values, which we were unable to reproduce here in a new patient population for AVP. Restrictively, it must be said that saliva values in ICU patients are fundamentally difficult to interpret because many of the drugs administered either cause dry mouth and make extraction difficult or cause hypersalivation which leads to dilution effects [21].

Another issue is the time of sample collection, which is assumed to influence hypothalamic neuropeptide concentrations especially in the central compartment [22]. However, studies on neurocritical care patients which mainly examined patients with aneurysmal subarachnoid hemorrhage, showed that AVP secretion neither in the blood nor in the CSF follows a diurnal rhythm [23, 24]. In our study on a more heterogenous patient collective, samples were obtained only during day shifts, where a steady level of light and noise prevailed at the ward to minimize potential external influences on AVP secretion.

The limited number of human studies using a wide variety of body fluids complicates the analysis of AVP concentrations. Moreover, AVP is elaborately and costly to analyze, which is the reason that some authors used the equimolar secreted copeptin as a surrogate for blood concentrations in their studies [25, 26]. This use of a further surrogate contributes even more to the heterogenous data situation. These circumstances prompted us to investigate AVP in the intensive care context in more detail.

The primary objective of our study was to examine AVP concentrations in the blood, CSF, and saliva compartments for any correlations to determine whether CNS concentrations in critically ill patients can be estimated by blood or saliva concentrations.

Our primary findings were only very weak to weak correlations between central and peripheral compartments in the entire patient collective. This confirms previous statements in the literature that central and peripheral secretion of neuropeptides are not necessarily coupled and that the blood–brain barrier is largely impermeable to endogenous neuropeptides [3].

The subgroup of postmenopausal women differed from the overall collective in showing a moderate positive correlation of AVP in plasma and CSF. This may have several causes: On the one hand, the coupling of central and peripheral AVP secretion mechanisms could differ between sexes. A rodent study for example showed that AVP plasma values increased in male and female rats with heart failure whereas mRNA levels were lower in females than in males [27]. Another reason might be that the permeability of the blood–brain-barrier is influenced by sex. Especially in the post-menopause, blood–brain-barrier function decreases due to a fall in estrogen levels whereas older men continue to produce estrogen from testosterone [28]. In the patient population we considered, we have to assume that all patients had a more or less compromised blood–brain barrier due to their underlying disease. However, a difference in disease severity between males and females cannot be suspected, because there was no significant difference in neurologic outcome.

In the second part of our study, we attempted to capture, at least roughly, the relationship between AVP and volume and electrolyte status. We chose serum sodium and hematocrit as two parameters from the routine laboratory that seemed suitable for this purpose. This choice based on the fact that sodium concentrations account for a large part of serum osmolality [29], and an older study by Murton and coworkers found significant correlations between AVP and sodium as well as hematocrit in critically ill patients with burns [30]. Hematocrit can be used in a common formula to calculate plasma volume [31].

Regarding the whole patient collective, only very weak to weak correlations could be shown between AVP concentrations in CSF, plasma, and saliva and sodium and hematocrit. This somehow reflects the findings of a historical study, where a rise in plasma AVP only after an acute salt load has been shown whereas 90 min after the salt load, no correlation could be found between plasma osmolality and AVP concentration [32]. Our patients did not have acute changes in osmolality and hematocrit, as patients with large volume shifts and acute blood loss were not included in the study.

Again, the subgroup of postmenopausal females differed from the whole collective. Postmenopausal women showed moderate inverse relations of AVP in CSF and serum sodium and hematocrit, where the correlation with sodium was statistically significant. This finding may be a hint to sex differences in volume and electrolyte regulation. At least in animal studies, plasma AVP response to hemorrhage was higher in female rats whereas pituitary AVP content was lower [10]. A human study showed that osmotic AVP release threshold is influenced by estrogen, and osmotic induced AVP release is reduced by sex hormones [33].

In rats with liver cirrhosis, differences between males and females in AVP release were also detected which provided an explanation for females not developing hyponatremia in that study [34].

Regarding outcome, no significant differences in AVP concentrations of the three compartments blood, CSF and saliva were found according to the Glasgow Outcome Scale (GOS) at discharge from ICU. However, our patient collective included only few patients with good neurological outcome, and the vast majority was GOS 3 which means severe disability and dependency in daily life [35]. Therefore, our patient collective was too small and imbalanced to draw conclusions. However, patients with good neurological status are often not in need of an extraventricular drain and are therefore not amenable to CSF sampling.

## Conclusions

Although no sex differences were found in absolute concentrations of AVP in central and peripheral compartments, there is discrete evidence of different secretion and distribution mechanisms in men and women. Our study should be considered a pilot study because of the small number of patients, especially premenopausal women. However, our results encourage further investigation of sex differences in central and peripheral AVP regulation that could account for both physical and psychological outcomes in critically ill patients.

## Data Availability

The datasets supporting the conclusions of this article are included within the article. Further information is available from the corresponding author upon reasonable request.

## Abbreviations

AVP: Arginine-vasopressin
CSF: Cerebrospinal fluid
GOS: Glasgow Outcome Scale
ICU: Intensive care unit
